# Applicability of a national strategy for patient-oriented research to people who use(d) substances: a Canadian experience

**DOI:** 10.1186/s40900-022-00351-z

**Published:** 2022-05-24

**Authors:** Bernadette Pauly, Ginger Sullivan, Dakota Inglis, Fred Cameron, Jack Phillips, Conor Rosen, Bill Bullock, Jennifer Cartwright, Taylor Hainstock, Cindy Trytten, Karen Urbanoski

**Affiliations:** 1 University of Victoria School of Nursing, Canadian Institute for Substance Use Research, Victoria, Canada; 2 University of Victoria, Canadian Institute for Substance Use Research, Victoria, Canada; 3 SOLID Outreach Society, Victoria, Canada; 4 Umbrella Society for Addictions and Mental Health, Victoria, Canada; 5 Victoria Division of Family Practice, Victoria, Canada; 6 BC Support Unit, Advancing Patient Oriented Research, Vancouver Island Regional Centre, Victoria, Canada; 7 Island Health Authority, Victoria, Canada

**Keywords:** Patient oriented research, Public involvement, Community based research, Substance use, Community engagement, People who use drugs

## Abstract

**Background:**

Europe and North America are in the grips of a devastating overdose crisis. People who use substances often feel unsafe to access healthcare due to fears of stigma, blame, judgement, poor treatment, or other repercussions. As a result, they often avoid, delay, or leave care, resulting in premature death and missed opportunities for care. Internationally, there have been concerted efforts to move towards patient-engaged research to enhance the quality of health care systems and services. In Canada, the Canadian Institutes of Health Research (CIHR) Strategy for Patient-Oriented Research (SPOR) initiative promotes engagement of patients as active partners in health care research. As part of a community based patient oriented research project, we critically analyze the SPOR framework to provide insights into what constitutes safer research with people who use(d) substances.

**Methods:**

We undertook a two-stage process that began with a review of community based research principles and the SPOR framework. At the second stage, we undertook a qualitative descriptive study employing focus groups to generate description of the adequacy and appropriateness of the SPOR framework for guiding research with people who use(d) substances on four key dimensions (patient engagement, guiding principles, core areas of engagement and benefits). The data were analyzed using qualitative content analysis to identify key issues and insights.

**Results:**

While the SPOR framework includes a range of patient roles, principles and areas for engagement, there are issues and gaps related to essential elements of safe patient-oriented research for people who use substances. These include an individualized focus on patients as partners, lack of recognition of community benefits, power imbalances and distrust due to systemic stigma, engagement as one way capacity building and learning, and lack of accountability for taking action on research findings.

**Conclusions:**

Given the extent of stigma in health care and the ongoing illicit drug policy crisis, strategies for enhancing equitable Patient-Oriented Research (POR) include shifting language from patient partners to community researchers, recognizing power inequities and adding trust and equity as core POR principles including pay equity. Employing community based participatory research as a POR methodology allows the lead researchers to fully engage community throughout the research process, enhances community benefits and accountability for action.

## Plain English Summary

In Canada, one of the lessons being learned as we navigate the current dual public health crises (COVID-19 pandemic and illicit drug overdoses and deaths) is the extent to which substance use and access to services is highly stigmatized, especially when combined with poverty, homelessness and perpetuated by racism and other forms of discrimination. Stigma and lack of feeling safe in health care can result in avoidance, delays or leaving care early, resulting in premature death and lack of essential care. Internationally, there has been a push to improve health services by involving patients as active partners in the research process. Termed “patient-oriented research” (POR), Canada’s primary federal funding agency for health research (Canadian Institutes of Health Research, CIHR) created the Strategy for Patient-Oriented (SPOR) Initiative to guide and support researchers involved in POR projects. As part of a POR project, our research team examined the SPOR Framework to determine its value in guiding research with people who use substances. Our team included people with lived and living experiences of substance use, academic researchers, health service providers, and decision makers. Five focus groups were held to review the SPOR Patient Engagement Framework and discuss strategies for successful POR. We found numerous gaps related to important elements of POR with people who use substances including the need to move away from ‘us and them’ on research teams and shifting away from individuals to communities, build trust, explicitly attend to power inequities between research partners, and acting on findings. These insights provide recommendations for more equitable POR research with people who use substances.

## Introduction

Europe and North America are in the grips of a deadly and ongoing overdose epidemic [1–5]. Between January 2016 and March, 2021, more than 22,000 Canadians died due to illicit drug poisoning [6]. In British Columbia (BC), Canada, rates of overdose deaths have been escalating since 2012, and in April 2016, the Provincial Health Officer declared a public health emergency which is still in effect today [7]. In 2021, in BC, there was a record 2224 drug toxicity deaths in which equates to an average of 6.1 per day and a 26% increase compared to 2020 [8]. Since the declaration of the COVID-19 public health emergency, overdose deaths due to a toxic drug supply have increased [9, 10]. COVID-19 has made the illicit drug market more unpredictable and increasingly toxic, pushed individuals to use in isolation, and impeded access to harm reduction and life-saving services [11, 12].

Substance use related stigma impacts people’s ability to access essential health care services [13, 14]. Stigma, stereotyping, and concerns of judgement and discrimination can lead people to delay, discontinue, or avoid care, resulting in missed care opportunities and ultimately premature death [15–19]. These experiences and fears increase in a context of social, economic, racial, and other forms of discrimination with power differentials between those seeking care and those providing care [20–22]. Illicit drug use, in particular, is highly stigmatized, especially when coupled with socio-economic disadvantages and/or diagnoses of HIV/AIDS or Hepatitis C virus—conditions that are themselves marginalizing [15, 21–23]. Stigma is ingrained in health care and social systems due to current policies that criminalize drug use and prominent narratives that people who use substances are responsible for their problems [14, 17]. In addition to impacting service use and health outcomes, stigma also leads to the exclusion of people who use substances from service design and research [24–26].

Patient-oriented research (POR) refers to actively engaging ‘patients’—individuals with personal experience of health issues and those close to them—in the design, implementation, and translation of health research to improve health services [27–32]. Involve was initiated in 1996 to promote active patient engagement in health and social science research. NIHR took over Involve in 2020 and identify community engagement and involvement as a core research component (See https://www.nihr.ac.uk/researchers/apply-for-funding/how-to-apply-for-global-health-funding/community-engagement-and-involvement.htm and https://www.invo.org.uk/) [31, 33]. The Patient-Centered Outcomes Research Institute (PCORI) in the United States funds research to investigate patient-oriented research in five priority areas, including health care delivery and disparities research [34, 35]. According to PCORI, researchers should include participants representing the spectrum of the population facing the health decision of interest [35].

In Canada, the Canadian Institutes of Health Research (CIHR) launched the Strategy for Patient Oriented Research (SPOR) to promote and support greater patient involvement in health research. The overall purpose of SPOR is to fund research that engages patients as partners, focuses on patient priorities, and improves patient outcomes, both individually and in communities [31, 32]. To support researchers with this initiative, CIHR developed the SPOR Patient Engagement Framework (hereafter referred to as the SPOR framework) [31, 32]. The SPOR framework is designed to address key concepts, guiding principles, and core areas for patient engagement to be adopted by all partners [31, 32]. As of 2016, more than $357 million (CAD, $289 million USD) has been invested in this initiative [31, 32, 36]. As with Involve and PCORI, the intention of SPOR is to improve health research that will ultimately improve health services [31–33, 35]. Framework development was through a consultative process that included a cross-representation of Canadian patients and patient engagement experts. The framework is intended to be a living document and re-evaluated based on experiences with implementation.

While POR, in general, refers to engaging a broad range of patient populations, researchers often do not successfully engage specific patient populations (often marginalized populations) in the research process [37]. Similar to other forms of health care research, people/groups are excluded from research due to stigma and marginalization [17, 37–39]. The purpose of this paper is to provide a critical analysis of the Canadian SPOR Framework in terms of its adequacy for guiding engaged, culturally appropriate, and safe research from the perspective of a research team co-led by people with lived and living experience of substance use. This research is particularly timely in the wake of a toxic drug poisoning public health emergency and calls for greater engagement of people who use drugs in responses and research. Based on this analysis, we provide insights and recommendations for enhancing the SPOR framework through identification of key principles and practices that can enhance equity and meaningful research partnerships with people who use(d) substances.

## Research with people who use substances

Substance use has been the subject of considerable academic research with the potential to intensify or reduce stigma directed toward people who use(d) substances. Substance use research, similar to other forms of research, has typically been conducted without the involvement of people who use(d) substances. Such models of research can intensify stigma as well as fail to address the key issues that are important to the population or a specific community [38, 39]. For more than three decades, drug user, and harm reduction movements have advocated for and promoted the involvement of people with lived and living experience in decisions that impact their lives, including research to enhance self-determination and well-being [40–44]. Research guidelines and frameworks have been developed by and for people who use(d) substances to promote more equitable, ethical, and effective partnerships [38, 40, 42–48]. The emphasis of these movements is to centre the experiences of people who use drugs to inform and guide research with the explicit intention of improving policy, practices, and services.

There are many issues to be worked out when conducting research in collaboration with people who use(d) substances, such as power differentials between members of the research team and the potential impacts on working relationships, participant compensation, training, and capacity building [44, 49, 50]. Notwithstanding the growing interest in patient engagement and the development of the SPOR Framework initiative, there are few examples of the inclusion of people who use(d) substances in POR and few discussions of the spaces and ways that practically and safely allow for inclusion in research [38, 39]. In our previous research, we identified cultural safety as a potential theoretical framework to guide our research project [51, 52].

## Cultural safety as a theoretical lens

The societal stigma faced by people who use(d) substances is particularly intense and multifaceted [14, 16, 22]. Many people who use(d) substances, particularly those who also experience structural disadvantages, have negative and traumatic past experiences with health care and research [20, 53–56]. Structural substance use related stigma, especially when combined with systemically produced discrimination (colonialism, racism, classism, and poverty or homelessness) and/or other marginalizing health conditions, is a barrier to involvement in health care research. People are often excluded from research or feel unsafe to participate. While these experiences speak to the need for greater patient involvement in research, there is little shared trust or basis for collaborative relationships given the historical and current context of stigma and criminalization of drug use.

Cultural safety is concerned with mitigating discrimination in health care for Indigenous people [57]. As a concept/philosophy, cultural safety draws attention to taken-for-granted norms, assumptions, policies, and practices in health care, education, and research [51, 57–59]. Developed by Maori nurse scholars in the 1990s to contribute to the more respectful care of Indigenous peoples in New Zealand, the concept has been applied to situations where there are power imbalances, stigma, and other forms of marginalization that affect Indigenous and non-Indigenous people [57–59]. Cultural safety requires researchers to acknowledge and accept that safety is defined by people who use substances as well as recognizing their own privilege and power differentials between health care providers, researchers, and people who use substances. We considered cultural safety to be an approach that could help identify power imbalances, cultural dominance, and structural inequalities within a collaborative patient-oriented research team [52].

## Research purpose and objectives

The purpose of this study was to inform the practice of safety in POR research with people who use(d) substances. This study was embedded in a larger project that aimed to define safe primary care for this population [52]. As part of the larger project, we conducted a critical analysis of the SPOR Framework to determine its relevance and applicability for research involving people who use(d) substances, academic researchers, health care decision-makers, and providers. Based on our work together as a research team, we provide real-time insights on the SPOR Framework from the viewpoints of partners actively engaged in a SPOR-funded initiative during a period of intense and ongoing harms related to stigma and overdose deaths.

## Research team

The research team was composed of academic researchers, community researchers with lived experience (people who use or have used substances), health care decision makers, and providers. The term community researchers was specifically chosen by the team members with lived experience as a better description of their role as active and engaged partners in the process of research rather than the term ‘patient partner’. The community researchers were affiliated with one of two local organizations that provide support, advocacy, and services to people who use(d) substances (SOLID Outreach or Umbrella Society for Mental Health and Addictions). Both organizations are peer-run, with a shared concern about substance use related stigma in society and a commitment to addressing harms of substance use. As per POR, these organizations would be considered patient partner organizations. Pauly, the Island Health Scholar in Residence, and Urbanoski, a CIHR Canada Research Chair, had established research relationships with Island Health, SOLID Outreach and Umbrella Society. Island Health Research Department (Trytten) played a key role in initiating this research and bringing together community and academic researchers along with Victoria's Division of Family Practice at the onset of the research, as well as providing practical and financial support in kind for a CIHR SPOR funding application. While not all of the parties had worked together before, all had a vested interest in a better understanding of how to safely conduct POR to improve primary care services for people who use substances.

## Research approach/methods

### Study design

As an approach to involving ‘patients’, we drew on principles of community based participatory research (CBPR) to guide the overall study. CBPR explicitly includes full participation of those impacted by inequities throughout the research process and prioritizes action [60]. CBPR is not a specific method per se but a collaborative approach to research which equitably involves those impacted by inequities in the process of generating knowledge from identifying the research focus, involvement in data collection, and analysis for the purpose of action [60]. CBPR outlines a specific approach to collaborative inquiry that is critical and action oriented with community at the centre of the research. CBPR aligned with a critical perspective, which includes the recognition of difference in power between community members and health system decision makers with clear commitments to the democratization of knowledge by privileging community members who are often excluded from research and decision making [60]. CBPR has been important to guide research with diverse groups, including people living with HIV/AIDS, street-involved youth, people who experience intimate partner violence, and people who use substances [17, 39]. Aligned with CBPR principles, our research team was specifically structured to ensure that people with lived experience were full and meaningful partners in all phases of the research. This was achieved by creating a core research group consisting of six people with lived or living experience, two lead academic researchers, and one academic research assistant (RA). This core group was responsible for design and implementation of the full study, with the wider group of health care providers and decision makers being involved as advisors to the core decision-making team.

## Materials and methods

Within an overall framework of CBPR, we used a qualitative descriptive design for this study. Qualitative description is useful when the aim is to describe a phenomena (who, what, when or where) rather than answer how or why questions from the perspectives of those who have experience with the phenomena [61, 62]. We chose focus groups as a method to explore the various aspects of the SPOR Framework [63]. Focus groups often consist of a small group of people and a range of participants according to the topic and the type of data being collected. Ideally, focus groups should include enough participants to generate breadth on the topic while having few enough to let everyone share in the discussion [63]. The data emerging from focus group discussions are used to understand a specific subject, which in this case were the views of health care providers, people who use or used substances, and decision makers regarding POR generally and the SPOR Framework in particular. Our approach involved two steps, detailed below. Ethical approval for the study was obtained from the University of Victoria and from Island Health, one of seven BC regional health authorities.

### Reviewing the SPOR framework, CBPR and cultural safety

The first phase of data collection focused on identifying processes and laying the groundwork for effective POR that would be mindful of the differences in power between team members. Community members were not required to have formal training in research to participate but were expected to have an interest in research and in collaborating with people who have different experiences and backgrounds to their own. In the first phase of the study, the co-principal investigators hosted two half-day meetings with the research team. These initial meetings aimed to introduce community-based participatory research and the CIHR SPOR Framework, as well as cultural safety principles and practices.

### Focus groups

The second phase consisted of three focus groups with members recruited from the full research team as a form of purposive sampling in which key informants have knowledge of the phenomena being studied, in this case patient oriented research. Consistent with CBPR, the academic researchers developed an initial draft of a semi-structured discussion guide that was reviewed by the whole team prior to the focus groups. The overarching question used to guide the focus group interviews was: “Is the SPOR patient engagement Framework adequate for all of us to guide culturally safe research with people who use substances?” The interview guide included further questions related to four concepts central to the SPOR Framework: patient roles, benefits, guiding principles, and core areas of engagements. The focus groups included community researchers with lived experience of substance use, academic researchers, health care decisions makers, and providers. The specific demographics of the 13 participants are not included in order to protect confidentiality but participants represented a range of ages from 20s to 60s of different genders with a wide range of incomes and employment situations including people on income assistance to those working as healthcare providers or holding leadership positions in healthcare. For the first two focus groups, participants were split according to their roles on the team. One group included six participants, and the other included seven participants. The third and final focus group discussion combined the two groups. The focus group interviews took place in a meeting room at one of the partnering organizations. Either lunch or snacks were provided for the participants, and people with lived or living experience of substance use received a cash honorarium of $25 as compensation for their time. Two female academic researchers and scientists from CISUR (Canadian Institute for Substance Use Research) (Pauly and Urbanoski) with experience in focus group facilitation led the sessions. Written consent was obtained from all participants before beginning and sessions lasted between 2 and 3 hours. During the discussion, the facilitators remained attentive to the difference in power between group members and encouraged the expression of diverse viewpoints. As well, opportunities for debriefing and follow up were available to all participants as needed to discuss any concerns emerging during the session. The discussions were audio-recorded, with the consent of participants, and transcribed verbatim by a trained research assistant with extensive experience in transcribing.

## Data analysis

Team members with formal qualitative research training analyzed the transcripts to identify key themes related to the use of the SPOR Framework key insights for guiding culturally safe POR (Pauly and Inglis). First, the analysts listened to audio recordings and read the transcripts several times to gain a sense of the initial codes relevant to each of the 4 dimensions of the SPOR framework. Thus, SPOR framework is used as an initial structure for organizing the data. Using methods of qualitative content analysis, data within and across codes was compared and contrasted to generate a set of initial and tentative themes [64]. The coding and themes were reviewed by another academic researcher on the team. To further enhance rigour, the tentative themes were presented to the full research team during the third focus group as a form of member checking. While key themes were affirmed, research team members highlighted where definitions of themes could be expanded or providing further examples to illustrate the findings. Following input from the team, these tentative themes were further refined to ensure data saturation in relation to the SPOR framework itself. We prepared this manuscript with reference to the COREQ (Consolidated Criteria for Reporting Qualitative Research) Checklist (http://cdn.elsevier.com/promis_misc/ISSM_COREQ_Checklist.pdf).

## Findings

Below we describe our learnings and reflections concerning key aspects of the SPOR Framework in a sustained CBPR study with people who use(d) substances. These insights are described in five themes: (1) Shifting roles: From patients to community researchers; (2) From individual to community benefits of POR; (3) Equitable partnerships and trust as key principles; (4) From engagement to drivers of research and co-learning; and (5) From safety to action in POR. Our findings are meant to help inform and enhance the development of POR projects involving people who use(d) substances. The findings are summarized in Table 1.

**Table 1:** Primary themes, gaps and recommendations

SPOR framework dimension	Theme	Issues/Gaps	Recommendations
*Roles*: patient as an inclusive term	Shifting roles: from patient to community researcher	Use of term patient implies passivity and lack of power Sets up an ‘us’ and ‘them’ dynamic Family as co-researchers lost in the framework	Shift away from use of term patient partner or researcher Use of the term community researcher Thread through families
*Benefits* for individual in terms of skills and knowledge	From individual to community benefits	Lack of recognition of community benefits	Use of CBPR Feeling like part of a Community (countering isolation) Feeling heard and benefits of research for a better future
*SPOR principles:* inclusiveness, support, mutual respect, and co-building solutions	Trust and equitable relationships as key principles	Lack of recognition of trust as key principle Lack of recognition of power inequities	Recognition of substance use related stigma and trust building Recognition of power inequities by all team members Ensure equitable pay Creation of a core research team with people with lived/living expertise
*Engagement* in governance and decision making, capacity building for patient engagement, and tools and resources	From patients to drivers and co-learning	Patients as ‘other’ rather than driving research Lack of recognition that researchers not just patients need capacity building	Use of CBPR Core Research Team Two way and co-learning
Missing SPOR component	From safety to action in POR	Missing emphasis on actioning the findings	Use of CBPR Accountability for action Catalytic validity

### Theme 1: Shifting roles: from patients to community researchers

The SPOR Framework defines ‘patients’ as “an overarching term inclusive of individuals with personal experience of a health issue and informal caregivers, including family and friends” [p. 5, 31]. Participants in the focus groups were asked to reflect on and describe ‘patient roles’ given their previous experiences in POR research. Focus Group participants stressed that the term patient implies passivity—whereby patients are passive agents invited to engage in research rather than full partners involved in all aspects of the study as per CBPR and their experiences. As one participant shared:

Yeah, like,,,patient is really pathologizing and implies a powerless sort of role compared to having what we would usually call community-engaged research. And then just...

some of this still continues some of that language. At the top of page seven, having the patient called “they” instead of “we” so it’s still coming from the researcher perspective here so instead of “they have mastered research skills” it should be “we have”—this could actually be written so that it is inclusive, to watch the language on “us” and “them”.

The term ‘patient’ is reflective of a power dynamic where the person who is defined as the patient has less power than others in the research process setting up a ‘we’ and ‘they’ dynamic. Additionally, many participants stressed that the language of the patient created a false binary of categories between people, where ‘patients,’ ‘researchers,’ ‘health care providers’ and ‘decision makers’ occupy exclusive groups. As one participant remarked: *“Anyone and everyone is a patient, whether they are researchers, people with lived experience, or providers.”*

There was consensus among the participants to replace the term ‘patient’ with a term such as ‘community researcher’ to capture more accurately the full and engaged role that people with lived experience can and do play in the process of research, from creating research questions to analyzing, interpreting, and presenting data such as was the case in this research project. Participants stressed that this semantic shift could help address the unequal relationships between the research team members and better acknowledge that ‘patients’ or people with lived experience are engaged as experts in the process of the research rather being objects of research or tokenized in the research process.

Given the definition of patient which includes families, participants reflected on the inclusion of family and friends in the definition. Participants decided that although the *family* is mentioned in the definition of patient, family is not threaded throughout the SPOR Framework. One participant stated,

There’s no mention in this [SPOR Framework] of specifically families, yeah, they include it but there is no how would that fit in, because I think families in this kind of setting is significant, they are ….significant contributors.

Like this participant, others highlighted that the focus on family in the SPOR Framework refers mostly to individual patients rather than a unit of people or chosen families as co-researchers. Participants appreciated the initial mention of family but felt it “got lost”. Overall, participants felt that more could be done to better articulate family as research partners. As the participant above notes, families are important contributors to substance use research.

### Theme 2: From individual to community benefits

The SPOR Framework outlines the benefits for ‘patients’ through their involvement in POR. For example, “Patients gain many benefits through their participation, including increased confidence and mastering new skills, access to information they can understand and use, and a feeling of accomplishment from contributing to research relevant to their needs” [31]. When participants were asked to reflect on the benefits of POR, and more specifically of using the SPOR Framework to guide the larger project*,* they shared that while they agreed with the benefits identified in the Framework, there were additional benefits that were not listed. As one participant with living experience of substance use shared:

I have benefited so much . . . from building relationships with everybody involved [all the different stakeholders involved in the project].

Another participant with living experience remarked:

A big one for me is learning all these different organizations, roles in the community, and networking with them . . . knowing all the resources available in the community. That’s a huge benefit to my involvement.

The SPOR Framework identifies other benefits of POR, including “improved health, improved access to the health care system, the right treatment at the right time, and improving the cost-effectiveness of the health care system.” Yet, POR is ultimately aimed at “achieving benefits that matter to patients” [p. 5, 31]. Several participants described the benefit of feeling like they are part of a community that counters feelings of isolation. They hoped that their involvement may inspire others to get involved when it becomes known that the research is meaningful and truly participatory. Participants shared that the relationships built in the larger project involving this research team were different than other “traditional forms of research” and described these relationships as ones that could “extend well beyond the project.” For example, many of the community researchers, health planners, and service providers have ongoing service relationships. As will be discussed later, the community involvement and benefits highlight the value of using CBPR as a specific approach to POR.

Focus group participants thought that the Framework failed to highlight “community benefits” that they saw to be an important part of POR. One participant with lived experience stated: *“*Ya it isn’t just like for me or us but this [research] can benefit many people.*”* Another person with lived experienced of substance use stated,

Research done this way gives us the feeling that our voices are actually heard and gives you a feeling that you help shape a future world that you’ll live in.

This statement is particularly meaningful in a context of substance related stigma where people who use substances are often stigmatized or tokenized rather than listened to in decision making processes. Participants expressed that POR “done right” with “true engagement” of all partners benefits the community because voices of people and families with lived/living experience are being heard through the research. In addition, when you involve people with a variety of different experiences, academic researchers, clinicians, and people with lived experience, the findings and knowledge products are more applicable and representative of a “group of people” rather than only experts.

### Theme 3: Equitable partnerships and trust as key principles

The SPOR Framework lays out the principles for engaging ‘patients’ in health research: inclusiveness, support, mutual respect, and co-building solutions. While focus group participants agreed with these principles, they also highlighted the importance of additional principles, namely building trust and creating equitable partnerships. Several participants shared that the principle of respect in the SPOR Framework is “good” but stressed that the principle of trust is critically important in POR with people who use(d) substances due to high levels of systemic distrust as consequence of stigma and past negative experiences in healthcare [65, 66] As one participant shared, “trust as a priority” was part of what made the current research project different:

Yeah, I would also like to see trust in there [SPOR Framework] somewhere because I think it’s part of the mutual respect but I think going back to … confidentiality and just you know, assuming that things will kind of be here, stay here, not go anywhere else, I think I’d like to see trust built in a bit more as well.

Trust was viewed as central to the development of productive research relationships. As one participant with living experience explained:

You’re coming to the table and basically displaying your life for people to see, so you need to trust that those people you’re giving information to are going to use it wisely and aren’t gonna distribute it around if they do not have to . . . and also trust on the other side too, that you build trust so that you can actually share that information to begin with regardless of where it is going to. But like, I’m not going to open up unless I trust people.

Participants shared that POR with people who use(d) substances has the potential to shift negative and stigmatizing stereotypes about drug use in health care to more positive images and understandings. Participants shared that within the context of the larger project, it was important to have an aim of equitable partnerships and recognition of differences in power among team members. As noted above, people with lived and living experience formed the core research team rather than acting as advisors or consultants in the research process in order to shift the power dynamics. This shifting from advisor to core research team members was a deliberate strategy to reduce power imbalances that often exist in research teams between community, academic, and health systems stakeholders.

Participants stressed that although the SPOR principles are adequate, with some additions, they are “only principles”—how the principles are taken up and upheld in the context of the research is what is most important. In other words, the ability to apply the principles is critical and how they are enacted in the process of the research is what matters. To illustrate the principle of equity, one participant spoke about the importance of pay equity and highlighted that in many research projects, “patients” or people with lived experience do not receive compensation that reflects the years of experience and expertise they bring to the research. In the larger project, community researchers were paid an hourly wage comparable to that of a graduate level researcher and amounted to an hourly sum that was above the living wage level for the region. However, the pay was not necessarily reflective of differences in the years of experience and expertise brought to the project by community researchers.

### Theme 4: From engagement to drivers of research and co-learning

The SPOR Framework outlines three key areas for engaging ‘patients’ in the research process: (1) engagement in governance and decision making, (2) capacity building for patient engagement, and (3) tools and resources. When asked about their impressions of three core areas of engagement, participants stressed that the Framework is written in such a way to put the patient in the role of the ‘other’ rather than leading and driving research. As one participant remarked when reviewing the SPOR Framework document:

I put a couple of – like on the last two bullets where it [SPOR Framework] says patient “as contributors” I would say “and key players” in identifying the right research question and then the bottom where it [SPOR Framework] says “as supporters of research study” should say “as drivers of research studies.

This participant is highlighting the importance of asking the right research questions, as well as conducting the research in safe ways with accurate interpretations.

Participants stressed that researchers and health care providers also need capacity building, not just ‘patients.’ They stressed that the document should define capacity building as “two-way” and highlight the co-learning that occurs among *all* members of the team. Several participants suggested that the language of the document should be rewritten to shift language from “them” to “us” to truly reflect a co-learning approach. A reciprocal relationship could help disrupt the power differentials that exist between academic researchers, health providers, and people with lived experience. This suggestion, and the need for actionable research, are further discussed below as part of culturally safe research.

### Theme 5: From safety to action in POR

Our study sought to create an understanding of what constitutes safe research for people who use(d) substances. Participants stressed that, in order for this aim to be met, the research must be led by people with lived experience of substance use (either past or current) within a collaborative and community-engaged Framework (i.e., only people with lived and living experience can determine what is considered safe). In other words, full participation throughout the process should be the standard to avoid tokenism on research teams. As one participant with lived experience noted,

Having peers involved makes it culturally safe because they are trusted members of the community. Peers are going to call you back, and make sure the facts are right.

In our study, community researchers were readily able to recruit and follow up with participants, which enhanced study participation but also ensured shared ownership of the research. As one participant remarked:

We (persons with lived experience) need to get to see the research that is published. We get to see what is written, and we get acknowledgment for our contribution.

In this way, there is accountability on the part of the whole research team to the community as well as acknowledgement of the contributions of community members.

Participants stressed that POR will be safe to the extent that all team members are aware of power imbalances between team members. As one participant expressed: “*There has to be equity first to have culturally safe research*.” Importantly, there has to be acknowledgement of the drivers of stigma for people who use(d) substances; without this acknowledgement “it is not—and cannot be—culturally safe research.

Finally, participants expressed their opinion that the SPOR Framework does not have a strong enough focus on action. Participants emphasized that, to be safe, the outcomes of the POR must be actionable and team members are accountable to each other for operationalizing these actions. For example, POR that aims to examine stigma within health care settings should also contain elements to reduce stigma and improve outcomes. Specifically, participants with lived experience stressed the need to ensure the research will be implemented—in other words, if the study does not get research evidence into policy and practice, it is “useless.” As one participant remarked,

The Framework is much too focused on the project itself and not enough on action, knowledge translation, or outcomes.

Some participants stressed that for patients and community groups who cannot hold research funds, seeing tangible outcomes from the research is essential. In other words, participants need to see change as a result of the work that they do; it is not simply enough to make recommendations. This focus on action is directly aligned and central to the aims of CBPR and key to operationalizing the hope that POR will improve health services.

### Strengths and limitations

This study was conducted in an urban center and involved people with lived/living experience with substance use with experience as community researchers in collaboration with decision makers and academic researchers. We had few participants who identified as non binary or Black, Indigenous or as a Person of Color. The peer led organizations had previous history and experiences with research as well as a history of working with the academic researchers on this research team. This is a strength in that there were previously established relationships. While this would facilitate criticism of the research framework, it may mean individuals were not comfortable voicing critical concerns about the research project itself. However, the focus was the SPOR framework not an analysis of this particular study which was reinforced during the focus groups. Further, throughout there was the potential for power dynamics to come into play particularly in the third focus group where all participants occupying different positions of power and authority were together in one group. In part, this was mitigated by having two focus groups separately first and having academic researchers who had well established relationships with both groups as facilitators.

## Discussion

POR seeks to engage a range of ‘patients’ in health care. Both PCORI and SPOR explicitly identify the importance of improving health and health services for those who are vulnerable to health inequities and/or disparities. In this POR project, guided by academic researchers, people who use(d) substances, health decision makers, and care providers came together to examine the adequacy of the CIHR SPOR Framework for informing substance use research with people who use(d) substances. Our findings reveal that while the SPOR Framework has strengths, it is not adequate to guide research in this area for several reasons. The Framework has an ‘us and them’ orientation, with patient partners constructed as passive and powerless with lack of specific attention to power imbalances in research relationships as well as a failure to include trust as a key principle. These are critical in the context of substance use research and structural stigma. There is little recognition of inequities that shape the lives of community members, the community benefits of research or the community as drivers of research as within SPOR patient partners are positioned as individuals to be engaged in research. We identified several key strategies that could be used to strengthen future POR with people who use(d) substances, including (1) shifting away from patient oriented language to community language; (2) the use of CBPR methodology to authentically engage members in all aspects of the research and increase accountability for action on findings; (3) development of trust and equitable relationships including ensuring equitable pay for community members; (4) development of a core research team composed of community and academic researchers to drive the research and mitigate power inequities; (5) recognition of opportunities for two way capacity building and co-learning and (6) the need to action research findings for benefit of the community. These ideas resonate with some of our previous analyses of SPOR and the lack of attention paid to key tenets of public health systems and services research [37].

Embedded in the SPOR Framework is language that unintentionally reinforces the ‘us and them’ divide and power inequities between people with lived experience, researchers, and health care providers and decision makers. We identified the need for a shift in orientation to ‘us and us’ so as to position people with lived experience on POR research teams as experts in their own right. This is critically important to achieve the aims of POR, specifically improving health systems and services. If the involvement of patients is central to research that improves systems, then as pointed out by participants, it is critical that a shift in power is needed where patients drive the research by identifying questions and determining how the research is conducted. CBPR is an important approach and methodology that can reorentate POR and enact more critically oriented POR.

In addition to this re-orientation, there needs to be explicit reconsideration of the term ‘patient’, as it is rife with power dynamics and fails to capture the important expertise that individuals, families, groups, and communities bring to the research as well as constructing patients as passive and powerless. This has particular meaning in the context of substance use and the exacerbation of neo-liberal narratives where people who use substances are seen as passive victims out of control, victims of disease of addiction or at fault for their drug use (e.g. moralizing) while ignoring drug policy and prohibition as harms of substance use and a source of inequities that are founded on racism and other forms of discrimination [51, 67, 68]. Thus, community partners have suggested we use the term community researcher or peer researcher to overcome some of these potential associations with patient.

POR with people who use(d) substances must be reflective of “true engagement” and attentive to power dynamics within research relationships. There is an already existing wealth and breadth of knowledge within the field of community-based participatory research that highlights strategies and approaches to address power dynamics within research relationships [38, 51, 52]. We specifically highlight that in much of CBPR, the focus is on partnering with people impacted by structural inequities [15, 38, 39] and the need for a shift in power dynamics between researchers and people experiencing inequities.

Given the frequent negative health care experiences of people who use(d) substances within an ongoing context of criminalization, the willingness to seek and access health services is often contingent on the development of trusting relationships [65, 66, 69, 70]. Absent from the SPOR Framework is recognition of the importance of building and establishing trust between research partners. This is especially critical for people who use(d) substances due to the extent of negative stigma and discrimination they experience in healthcare that intersects with racism and other forms of systemic discrimination [20,71–73].

Building trust and relationships is central to ensuring safety in research for people who use(d) substances. In this research, we had an aim of greater understanding of culturally safe research and began by drawing on previously developed concepts of cultural safety. Cultural safety was originally developed to address systemic racism and discrimination of Indigenous people [74,75]. Some have proposed it is relevant for other groups experiencing marginalization [57]. Based on our work, we feel that using the language of cultural safety to refer to safety for people who use(d) drugs, may take away from the importance of addressing Indigenous specific racism. So, in the context of drug related stigma, we prefer to use the term safety. The aspect of cultural safety that we found so valuable in our work was the principle that safety is defined by the service user and the recognition of differences in privilege among team members. More conceptual and theoretical work is needed to determine how to best capture the intention and importance of providing services and settings free of drug related stigma and the intersection with other forms of stigma.

However, little has been written about the process of building trusting research relationships within the context of POR. Developing trust requires establishing relationships that are based on human connection and equal exchanges—recognizing that all members of the research team bring unique and distinct skills, knowledge, and expertise to the research process. In our work, research relationships with people who use(d) substances were built over many years. Hence for people who use(d) substances, and populations where there are high levels of distrust, it is important to recognize that trust and building of relationships have to be included as important principles for meaningful research processes within POR and recognition of the time to build such relationships.

Our efforts align with other approaches to the conduct of ethical research with people who use substances. For example, a group of community-based researchers working in a highly marginalized and heavily researched community in Vancouver, Canada came together with thirteen representatives from peer-based organizations in the community to develop RESEARCH 101: A Manifesto for Ethical Research in the Downtown Eastside (DTES). This manifesto is based on the collaborative work of academic researchers, peer run organizations, and DTES community members (people with lived and living experiences). Similar to our findings, they suggest incorporating principles of CBPR into research processes with people who use(d) substances, specifically attending to power differentials between and among participants, and to establishing trust and meaningful reciprocity [38].

However, findings generated through CBPR with people experiencing inequities are often disconnected from service providers and decision makers. At the same time, there has been a focus on knowledge user-driven research which aims to engage health decision makers and providers but excludes service users or community members [76]. We see POR as having the potential to bring three groups together: people with lived expertise, academic researchers, and health decision makers and providers. However, this requires reconfiguring the relationships between these groups to address power dynamics and recognize people with lived experience as valued and knowledgeable community researchers. In our research we did this through the establishment of a core research team composed primarily of people with lived expertise, with researcher support and knowledge user partners who acted as advisors with their roles clearly delineated to be operationalizing the research.

If POR is intended to focus on priorities that are important to “patients” and produce information that is used directly to improve healthcare practice, therapies, and policies, a strong foundation for ensuring successful collaborations must be laid. Such a foundation must ensure that patient or community partners are equitably engaged and active in the governance, priority setting, and conduct of research and knowledge translation [31]. In our project, community researchers were key members of the core research team conducting data collection, analysis and interpretation. Together, academic and community researchers from the core team co-developed and co-presented the findings of the research with community members speaking to the process of how the research was conducted as well as providing specific examples and experiences to illustrate key findings. These were then shared with other decision maker partners.

Further, as highlighted by participants, POR research should have an action orientation. This need for action was identified as a gap in the SPOR Framework. Marginalized communities often face inequities including a lack of power and resources that make their time extremely valuable and their participation in research has value when there is relevant action as an outcome. In discussing action research, Lather identifies catalytic validity as a criterion for rigour [77]. Catalytic validity focuses on evidence of transformation or action (for example as policy or practice changes) as a measure of the research quality and for people to see that their time has been well spent. In this project, we developed a postcard and plain language bulletin as knowledge products, which were widely distributed locally by research participants and community members (www.spc.cisur.ca). Anecdotally, several community members reported using the postcard to advocate for their own primary care. In addition, team members with lived and living experience noted that the project contributed to the community through the opportunities they had to be heard at tables with regional health planners and service providers. Our team received a grant to support further knowledge translation, which was used to develop videos, a facilitator guide, and a workshop on reducing stigma and enhancing safety in primary care. The BC Support Unit also identified our project as a key exemplar of POR and profiled the research through its network (e.g., at conferences and in bulletins). Operationalizing and taking action on these findings was and continues to be a high priority in the context of dual public health emergencies in which COVID-19 has been escalating overdose deaths.

In the context of societal stigma, pay equity is a tangible symbol that recognizes the expertise of people who use(d) substances in respectful ways to build trust and collaborative relationships. In essence, it says we see your expertise and value it. This contributes to equity but also to respect and dignity. The BCCDC Paying Peers Guide provides specific guidance on how to pay people so as not to increase inequities, outlining some of the challenges and hurdles that need to be addressed [78].

Missing from the CIHR SPOR Framework is guidance surrounding the completion of research projects and future engagement. Investing in long term relationships signifies continuity and maintains confidence among all parties, but in particular between academic researchers and community participants. Often at the end of the research the benefits for academic researchers are apparent (e.g., publications, policy implications, future grant funding opportunities), but the benefits to the community and non-academic partners are often less clear. Thus, academic-community relationships often can and do continue to address community priorities.

## Conclusion

We have highlighted insights from a reflection on the SPOR Framework and the limits of this framework for guiding research related to substance use in health care research. While the Framework has a number of strengths, its overall “us and them” orientation locates community members as patients and ‘others’ as opposed to them being a driving force in research. The failure of the Framework to recognize power imbalances and trust as a principle limits its value for working with communities that experience inequities due to substance use related stigma. Shifting patient oriented language, use of CPBR, creation of a core research team as well as community researcher roles that drive the research with greater recognition of substance use stigma among all team members, an emphasis on co-learning, two way capacity and accountability for action would greatly improve the applicability of the SPOR Framework for use with people who use(d) substances. In turn, this would ensure achievement of the goals of SPOR related to improving the quality and equity of services and patient outcomes for people who use(d) substances.

## Data Availability

Given the qualitative nature of the project and issues related to confidentiality, transcripts are not available but interview guides are available upon request.
